# The Effect of the Length and Distribution of Implants for Fixed Prosthetic Reconstructions in the Atrophic Posterior Maxilla: A Finite Element Analysis

**DOI:** 10.3390/ma12162556

**Published:** 2019-08-11

**Authors:** Brunilda Gashi Cenkoglu, Nilufer Bolukbasi Balcioglu, Tayfun Ozdemir, Eitan Mijiritsky

**Affiliations:** 1 Department of Oral & Maxillofacial Surgery, Faculty of Dentistry, Albanian University, 1001 Tirana, Albania; 2 Department of Oral Implantology, Faculty of Dentistry, Istanbul University, 34093 Istanbul, Turkey; 3 Department of Otolaryngology, Head and Neck and Maxillofacial Surgery, Tel-Aviv Sourasky Medical Center, Sackler Faculty of Medicine, Tel-Aviv University, 5219100 Ramat Aviv, Israel

**Keywords:** dental implant, finite element analysis, atrophic maxillary sinus, prosthodontics, stress, overload

## Abstract

In this study, different prosthetic designs that could be applied instead of advanced surgical techniques in atrophic maxilla were evaluated with finite element analysis. Atrophic posterior maxilla was modeled using computer tomography images and four models were prepared as follows: Model 1 (M1), two implants supporting a three-unit distal cantilever prosthesis; Model 2 (M2), two implants supporting a three-unit conventional fixed partial denture; Model 3 (M3), three implants supporting three connected crowns; and Model 4 (M4), two implants supporting two connected crowns. Implants 4 mm in width and 8 mm or 13 mm in length were used. A linear three-dimensional finite element programme was used for analysis. The maximum principle stress (tensile) and minimum principle stress (compressive) were used to display stress in cortical and cancellous bones. The von Mises criteria were used to evaluate the stress on the implants. M1 was found to be the most risky model. The short dental arch case (M4) revealed the lowest stresses among the models but is not recommended when one more implant can be placed because of the bending forces that could occur at the mesial implant. In M2 and M3, the distal implants were placed bicortically between the crestal and sinus cortical plates, causing a fall of the stresses because of the bicortical stability of these implants.

## 1. Introduction

Implant therapy based on the principle of implant osseointegration has been well documented and is widely accepted. Despite high dental implant success rates, biomechanical risk factors tend to compromise their long-term success [1,2]. 

Bone quality and quantity, the number and size of implants to be placed, and the masticatory and parafunctional habits of the patient are a few risk factors that come to mind [3,4]. The posterior areas of the maxilla and the mandible are subject to higher forces of mastication. Furthermore, failure rates in the maxilla are reported to be higher than the mandible [5]. Particularly in the posterior maxilla, increased masticatory forces, as well as low bone density and decreased bone volume, compromise the placement of implants and increase the risk of failure [6]. 

Pneumatization is the process of maxillary sinus growth that continues throughout life. That said, the degree of pneumatization is one of the main factors leading to limited bone volume [7]. In addition, age, gender, dental status, cause of tooth loss, metabolic diseases, and smoking may significantly reduce the available bone height as well as bone quality in the posterior region [8,9]. A study involving 431 partially edentulous subjects revealed that the posterior existing available bone height was ≥6 mm in only 38% of maxillae [10].

Several surgical treatment options, such as guided bone regeneration and sinus augmentation surgeries, have been used to allow implant placement in the posterior maxilla [11,12]. Although these protocols have obtained a level of success, many patients may reject them because of the multiple surgical procedures, high costs, and longer treatment durations. The use of short implants (< 8 mm long) has been proposed as an alternative to these advanced surgical techniques, [13]. Recent reports [14,15,16,17,18,19,20] suggested that short implants may reach the same level of success as longer ones. The success of short implants is related to many factors such as oral hygiene, cantilever, implant-crown ratio, surgical technique, and implant diameter [14,15,16,17,18,19,20].

To enhance clinical success, it is necessary to understand how the stress concentration on implants is affected by the prosthesis type used. The use of finite element method (FEM) in implant biomechanics analysis offers many advantages over other methods in simulating the complexity of clinical situations. It allows researchers to predict the stress distribution between implants and the cortical or cancellous bone. Many previous finite element studies have examined the effect of occlusal forces on the implant placed in the different types of bone using finite element analysis [21,22]. 

The aim of this study was to determine the relative contribution of changes in prosthetic design supported by non-axially placed short and long implants in different localizations, of the atrophic posterior maxilla using a 3-dimensional finite element analysis. 

## 2. Materials and Methods 

### 2.1. Model Design

Cone-beam computed tomography [CBCT) (ILUMA Orthocad, Imtec Imaging, Oklahoma City, OK, USA) images of a partially edentulous adult patient taken from implant treatment planning records were used to form a geometric model of the atrophic posterior maxilla exhibiting severe maxillary sinus pneumatization. The patient provided written consent to the use of CBCT images in the study. The use of patient data was carried out in accordance with the policies and procedures of the Istanbul University, Faculty of Dentistry, Department of Oral Implantology. A total of 601 cross sections were obtained at 120 kvp, 3.8 mA, in 40 s. The sectional model scanned from the posterior maxilla was then transferred to a graphical preprocessing software (Rhinoceros 4.0, Robert McNeel & Associates, Seattle, WA, USA) which generated geometric configurations of the model, nodes and elements for a finite element program (Algor Fempro, Algor Inc., Pittsburgh, PA, USA) was used to construct the mathematical models consisting of bone, 2 or 3 osseointegrated implants, and the fixed partial dentures. A total of 4 models were prepared in accordance with implant plans which are frequently preferred in the clinic. The implants were placed at a 20° angle to the sagittal plane and 20° angled abutments were used. 

**Figure 1:**
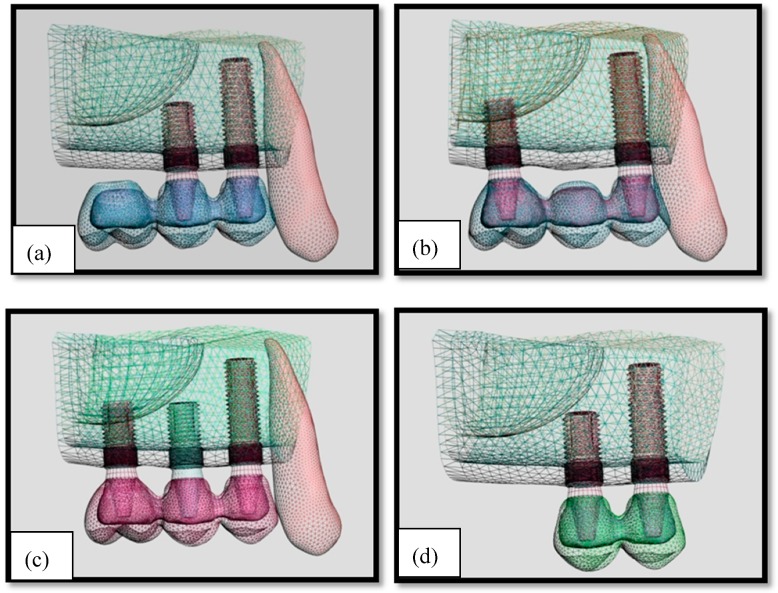
Implant distributions in models. (**a**): Model 1; (**b**): Model 2; (**c**): Model 3; (**d**): Model 4.

The number of implants and type of superstructure varied according to the model, as follows:

- Model 1: Two implants supporting a three-unit distal cantilever prosthesis (M1) (Figure 1a)
- Model 2: Two implants supporting a three-unit conventional fixed partial denture (M2) (Figure 1b)
- Model 3: Three implants supporting three connected crowns (M3) (Figure 1c)
- Model 4: Two implants supporting two connected crowns (M4) (Figure 1d)

The cylindrical 4.0 wide and 8-mm long or 4.0 wide and 13-mm long dental implants (AstraTech, Mölndal, Sweden) and 4-mm long 20° angled abutments were scanned using Nextengine Laser Scanner (NextEngine, Inc., Santa Monica, CA, USA). The 8-mm short implants were used in the 2nd premolar and first molar regions, while the 13-mm implant was used in the first premolar regions. Copings and crowns were also modelled using the Rhinoceros 4.0 (Robert McNeel & Associates, Seattle, WA, USA) preprocessing software. The crowns’ geometry and mesio-distal parameters were modelled in accordance with the description by Wheeler [23]. A minimum distance of 3 mm between adjacent implants and 1.5 mm of crestal bone in their buccal and palatinal aspects were conserved.

### 2.2. Material Properties

The bone was modelled as a cancellous core surrounded by a 1.5 mm thickness of cortical bone at the crestal region and a 1.5-mm thickness of cortical bone at the inferior border corresponding to the maxillary sinus. Chromium–cobalt alloy (Wiron 99; Bego, Bremen, Germany) was used as the lower structure and feldspathic porcelain was used as the upper structure (Ceramco II; Dentsply, Burlington, NJ, USA).

The metal thickness was prepared as 0.8 mm and the porcelain thickness was prepared 2 mm at minimum, considering final crown dimensions. All materials used in the models were considered to be isotropic, homogenous, and linearly elastic. The elastic properties used were taken from the literature (Table 1).

### 2.3. Interface Conditions

The FEM assumed a state of optimal osseointegration. The cortical and trabecular bone were assumed to be perfectly 100% bonded (osseointegrated) to the implant. It was assumed that there was an uninterrupted contact between the cortical bone and the trabecular bone, the dental implants and the maxilla, the implants and the abutments, and the abutments and the implant-supported porcelain fused to metal crowns. The cement layer was ignored. 

### 2.4. Element and Nodes

Models were meshed with 10-node brick elements. Considering the model type, the maximum number of elements permitted by the programme was selected to obtain realistic results. The number of elements and nodes in each model was as follows: 243.986 elements and 44.589 nodes in M1; 222.633 elements and 48.023 nodes in M2; 248.634 elements and 48.958 nodes in M3; and 171.726 elements and 31.668 nodes in M4.

### 2.5. Constraints and Loads

Axial, oblique, and horizontal loading were performed in all the teeth with the ratio reported in the investigation by Koolstra et al. [24] Loading regions were determined by occlusal contact points, as reported by Okeson [25]. Forces were applied at 2 points, 100 N vertically, 30 N horizontally, and 200 N obliquely on premolars. On molars on the other hand, forces were applied at 4 points, 140 N vertically, 45 N horizontally, and 280 N obliquely. 

Models were constrained in all directions at the nodes at the mesial and upper edges of the maxilla. Upper and lower prosthetic parts, implant screws, and bone tissues were harmonized using the Boolean method in the Rhino software and then force was applied.

### 2.6. Finite Element Analysis

The linear three-dimensional finite element programme (AlgorFempro, Algor Inc., Pittsburgh, PA, USA) was used for stress analysis. The maximum principle stress (tensile) and min principle (compressive) stresses were used to display stress in cortical and cancellous bone. The von Mises criteria were used to evaluate implants. Figures were obtained from the buccal and palatinal aspects of implants and from occlusal aspects of the maxilla to evaluate the stress distributions. The highest stresses were determined for axial, oblique, and horizontal loading. Evaluation scales were between 0 and the highest value.

## 3. Results

The maximum von Mises (MPa), the minimum-maximum principle stress (MPa) values, and displacement values (mm) are shown in Table 2, Table 3, Table 4 and Table 5, and their distribution is shown in Figure 2, Figure 3, Figure 4, Figure 5, Figure 6, Figure 7, Figure 8, Figure 9, Figure 10, Figure 11, Figure 12 and Figure 13 respectively.

### 3.1. Evaluation of the Models

#### 3.1.1. M1 Model

In model M1, owing to the cantilever design, the maximum von Mises stresses around implants were observed at the disto-vestibular region of the distal implant. Maximum principle stresses were observed on the mesial neck region of the mesial implant at the cortical bone, while at the cancellous portion of the bone they were observed at the facial neck region of the distal implant. However, the minimum principle stresses were observed at the distal-facial neck region of the distal implant at the cortical level and at the apical region of the distal implant at the cancellous bone level. During the displacement, rotation was observed in the horizontal and vertical planes. Moreover, the displacements were observed coronally and facio-distally. The cantilever demonstrated a sinking motion along with the distal implant, and the distal implant functioned as a fulcrum.

#### 3.1.2. M2 Model

In the second model, a conventional three-unit partial prosthesis is supported by two implants. The distal implant was placed bicortically between the crestal cortical and sinus cortical plates. Because of this bicortical support, the stress values at the mesial and distal implants were measured to be similar. Importantly, however, greater von Mises stress values at the distal neck region of the distal implant, at the implant-abutment junction specifically, were detected. Maximum principle stresses were calculated at the mesial cortical region of the mesial implant neck and at the mesial cancellous region of the same implant. Minimum principle stresses were valued at the vestibular region of the three implants in cortical level at apical region of the distal implant at the sinus cortical plate level and because it was supported by the sinus plate, the greatest value seen in cancellous value is seen at the disto-apical of the central implant. The displacement was observed in a disto-facial and coronal direction. 

#### 3.1.3. M3 Model

In the M3 model, three splinted fixed prosthetic crowns are retained by three implants. At the molar region, the implant was placed bicortically. In spite of being a short implant, the stresses seen in and around this implant were almost identical to the other implants. However, the implant is located at the molar region, which is subject to greater forces than the premolar region. Therefore, a slight difference of stress values greater than the mesial implants exists in this design. Stresses around the central implant were lower than those around the other implants. Thus, the absence of the central implant in M2 did not strongly influence the stresses and displacement values when compared to M3. The distribution of stresses and displacement was also similar to M2. 

#### 3.1.4. M4 Model

In this model, two splinted fixed prosthetic crowns are supported by two implants, and a short dental arch (SDA) was employed. The second premolar implant is short, whereas the first premolar implant is long. Because of the difference in length of these implants, the mesial implant demonstrated tensile stresses at the mesial neck region and a bending displacement toward the distal was expected in this implant. This bending motion would reflect on the tensile stresses of the cortical bone plate at the same mesial region. The greatest values of compression stresses were seen at the apical region of the short implant. Despite expectations of instability in this prosthetic design, when comparing the values of this model with others, much lower values were observed in this model because of the small values of forces exerted—owing to the crowns being premolars. In this model, there were no molar crowns, thus, no doubling of the forces occurred here. 

### 3.2. Evaluation of von Mises Stresses Occurring in Implants

On vertical, horizontal, and oblique loading, the highest von Mises stress value was measured on the second premolar of the M1 model where a distal cantilever is extended (Table 2). Instead of a distal cantilever design, a three-unit bridge constructed with a pontic corresponding to the second premolar site was shown to decrease the von Mises value by 1/3. The lowest value was measured at the M4 model.

### 3.3. Comparison of Models

In all the models, maximum tensile stresses and minimum principle values were found to be higher in the cortical bone than in the trabecular bone (Table 3 and Table 4). Moreover, the highest values were obtained on oblique loading. The M1 model with a distal cantilever showed the highest tension values at all three loading types. From a clinical perspective, when planning a three-unit bridge supported by two implants, it would be more advantageous to place a pontic between two implants instead of extending a distal cantilever. Posterior edentulous regions may be rehabilitated with three instead of two implants to facilitate a more balanced distribution of masticatory forces and decrease the loads transmitted to the jaw bones and implants.

### 3.4. Evaluation of Displacements Occurring in Bone

In models M2, M3, and M4, axial loads acted along the longitudinal axis of the implants and perpendicular to the long axis of the bone. They caused bone compression at the bottom and shear stresses at the lateral interface of the implants. Prosthesis and implants were displaced in an almost vertical plane, but not entirely vertically, because of the short implant used in the 2nd premolar and molar regions, which tend to displace the prosthesis a bit distally. Bucco-lingual loads caused tilting of the prosthesis and implants towards the buccal, yielding compressive and tensile stresses in the cortical bone around the neck region of the implants, on the buccal and palatinal sides, respectively (Table 5). 

## 4. Discussion

In this study, by using a 3D finite element analysis method, four different types of fixed prostheses models supported by implants (M1, M2, M3 and M4) were compared in terms of stress distribution in the maxillary posterior segment.

A segment of bone was modeled to simulate the posterior region of the maxilla. Its size was chosen so that the end effects (stress extended to the ends of the bone segment) would not impinge on the results at the region of interest. In a 3D FEA study, Sato et al. [26] reported that variations in the bone stress around an implant were negligible if the length of bone between the implant and the segment end was at least 4.2 mm. In the present study, this length was at least 10 mm, and even longer at the distal end. Moreover, loads and restraints were simplified, since only part of the maxilla was modeled. Although this simplification could be expected to bring about quantitative changes in the results, it was not expected to influence them qualitatively. The locations of loads (force) and restraints (reaction) were reversed from that of the real situation, but this reversal should not have significantly affected the results, since a force and its reaction have the same value. 

Average forces were used to calculate the stress in the bone. Since all the materials in the models were linearly elastic, the stress increased proportionally with the load. The bone was considered to be linearly elastic, homogeneous, and isotropic, and the implants were considered to be rigidly bound to the bone over their entire surface. Since the reality is more complex than this simulation, a qualitative comparison among models is advisable rather than focusing on quantitative data from FEA.

Following the loss of natural teeth, bone resorption in the maxillary ridge is expected. As a result of this resorption, implants generally need to be placed in a more palatal position. Therefore, to compensate, angled abutments are frequently used to connect implant-retained prostheses. It is for this reason that palataly-orientated implants and 20° angled abutments were preferred in this study. Tian et al. [27] stated that in cases where implants could not be placed in ideal positions, using angled abutments reduced stress values. Bölükbaşı et al. [21], on the other hand, reported that abutment angles, connected on to implants placed in ideal or other positions, as determined by FEM analysis, could affect stresses exerted on peri-implant bone. 

The results of this analysis concur with the findings of previous studies that used different investigation methods; therefore, the model employed in this study is considered to satisfactorily simulate reality. The tendency of stress concentration around the implant neck, which was evident in all of the models, was consistent with other results from the FEA of loaded implants, as well as with findings from in vitro and in vivo experiments and clinical studies, which demonstrated bone loss initiating around the implant neck. 

In the study by Tepper et al. [28], using the 3D FEA method, it was found that in the models where sinus cortical bone was eliminated, the values of the stresses were three times higher than the values of the stresses found at the models with sinus cortical bone modeled. In order for the results of this study to be more realistic, the maxillary sinus was modelled, and the apical parts of the implants placed at first molar area in the M2 and M3 were modelled to imitate contact with the base of the maxillary sinus. 

In all the models, the highest stresses occurred within the implants. These were due to the higher modulus of elasticity of titanium which peaked at 361 N/mm^2^ when applying the force of 280 N, the maximum force per crown in this study. As titanium alloys are known to tolerate stresses of up to 900 N/mm^2^ without irreversible deformation, this force applied to the system in this case was unlikely to cause implant failure [28].

In order to reproduce contacts that occur during mastication, in the current study, premolar and molar crowns were loaded on two and four points. Loads were applied vertically, horizontally, and obliquely (45°). The total vertical, horizontal, and oblique forces applied were 340 N, 105 N and 680 N. In our study, the ratio Fh:Fv:Fo = 1:3.5:7 among vertical, horizontal, and oblique loadings was used. This ratio, as referred to in many studies [28,29], was described by Koolstra et al. [24] in a study examining the maximum possible bite forces generated by means of three-dimensional mathematical model analysis. On the other hand. the points loaded by the forces were determined using Okeson’s [25] description of occlusion contact points. Masticatory forces vary from person to person, and it must be taken into consideration that increasing masticatory forces result in increased stresses at implants and the surrounding bone. 

Under vertical and buccolingual loads, stresses in M2 and M3 were higher in the cortical bone mesially to the mesial implant and distally to the distal implant than between implants. In M3, stress around the central implant was lower than around the other implants. In the linearly elastic materials, the stress values correspond to the gradient of displacement, namely deformation. Because the implants were rigidly anchored into the bone and the bone volume between implants was rather lower, the bone between implants, along with the implants, was almost uniformly displaced. Thus, this small bone deformation resulted in a low stress in the bone between implants. For the same reason, a lower stress was found around the central implant in M3. In contrast, at the mesial part of the mesial implant and at the distal part of the distal implant, the surrounding bone volume was greater, and it opposed a higher resistance to the action of the loads. Therefore, the bone close to the implants was more displaced than that located further away, and higher stress was concentrated near the implants. Under vertical and buccolingual loads, the bone around the central implant in M3 bore only a small amount of stress compared with the bone around the other two implants. Thus, the absence of the central implant in M2 did not strongly influence the maximum Von Mises stress and max and min Principle stresses, and similar values were found for M3 and M2 under vertical and buccolingual loads. However, under BL loads, bone around the central implant in M1 bore a degree of stress comparable to that around the other two implants. Therefore, the absence of the central implant in M2 determined substantial increase of the maximum von Mises stress compared to the value in M3 under BL loads. In M1, the high stress around the distal half of the distal implant, which was found under vertical loads, resulted from both the rotation in the vertical plane and the load applied to that implant. The rotation acted to extract the mesial implant, but this action was cancelled by the vertical load applied to that implant. Thus, almost no von Mises stress was found around the mesial implant. Under BL loads, the deformations that occurred as a combined effect of the rotations in the transversal and horizontal planes yielded increased stress around the distal implant and lower stress around the mesial implant. As predicted by Skalak [30], bone stress was greater under lateral loads than under axial loads. The rotation induced by the BL and Horizontal loads was responsible for the higher values of the maximum Von Mises stress under these loads. In all the models, higher bone stress was calculated under the BL loads than under horizontal loads. This was the result of the greater gradient of bone displacement under BL loads than under horizontal loads. In light of the high stress calculated for BL forces in each model, when planning and fabricating a superstructure, it is important to create an occlusal shape that minimizes lateral force components. The same principle should be considered during the occlusal adjustment.

Upon analyzing the literature, only a few studies were found using a similar methodology. De Souza Batista et al. [31] studied stresses generated at the abutment, implant, and fixation screw levels, and stresses and strains on the trabecular and cortical bones by preparing three-unit prostheses designed with pontics in the posterior maxilla and a mesial and/or distal cantilever. Three-implant-supported prostheses were prepared as part of the first model. The second model was composed of two implant-supported prostheses with a central pontic and the third model consisted of two implant supported-mesial cantilever restorations. Finally, the fourth model was two implant-supported three-unit bridges with distal cantilevers. An axial force of 400 N and 200 N oblique masticatory force were applied. The results obtained were similar to those of our study. Clinically, the most reliable model is the first model. For cases where two implants are planned, the central pontic model is proven to be more advantageous. Cantilever extended prostheses are classified as the highest-risk model. In the event that a cantilever design is planned, a mesial cantilever extension instead of a distal one is suggested. Similarly, in our study, the highest-risk model was determined to be the 1M model, with a distal cantilever. Also, it follows that in the cases where a two-implant-supported three-unit bridge is planned, the design describing a central pontic should be preferred. The design with a three-implant-supported three-unit bridge was found to be more advantageous when compared to the first two models, and was found to carry similar risks to a two-implant-supported two-unit bridge constructed at a posterior maxillary region with insufficient bone volume. 

Küçükkurt et al. [19] compared alternative treatment options, placing implants in cases of inadequate bone volume in posterior maxilla. Five 3D models, including a three-unit fixed prosthesis were modeled: a control model (two implants with medial pontic), lateral sinus lifting (LSL; 6-mm graft was created in the sinus, and an implant 10 mm in length was placed on the first molar position. An implant was also positioned in the first premolar site), a short dental implant placement (SIP; 5 mm in length implant was placed in the first molar tooth region and 10 mm in length implant was placed to first premolar tooth region), a tilted implant placement (TIP; 45° mesially tilted implant was placed to first molar site and standard implant placed to first premolar site) and a distal prosthetic cantilever (DC; 2 implants were placed to premolar sites). Vertical and oblique forces were applied. According to study results, the LSL method should be the first choice among treatment options. The SIP method may be preferable to the TIP method. The worst stress values were obtained in the DC model. In our study, the aim was to evaluate different treatment options where sinus lifting surgery was not intended. Therefore, we did not create a grafted sinus model.

## 5. Conclusions

Cantilever implant prosthesis (M1) may induce high bone stress, while connected crowns, supported by three implants (M3), may induce low bone stress. Furthermore, under a load with predominant axial components, a conventional fixed partial denture supported by two implants (M2) may create bone stress comparable to that calculated for connected crowns supported by three implants (M3). Two-crown-connected prosthesis (M4) used in short dental arch cases showed the lowest stresses between the models, but is not suggested when one more implant can be placed because of the bending forces that can occur at the mesial implant. Bicortical fixation would cause a fall of the stresses because of the stability of the implant. By preparing shallow cusps at the occlusal table, the forces would be transferred more axially to the implant, thus, lower stresses would occur. However, in occlusions with large buccolingual force components, only the connected crowns supported by three implants may minimize the harmful effect of these loads.

## Figures and Tables

**Figure 2:**
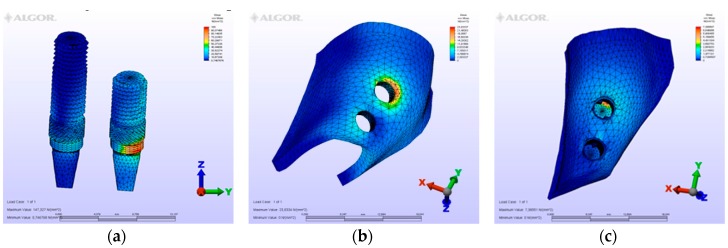
Model M1, axial loading. (**a**) the maximum von Mises stresses around implants; (**b**) maximum principle stress in cortical bone; (**c**) maximum principle stress in cancellous bone.

**Figure 3:**
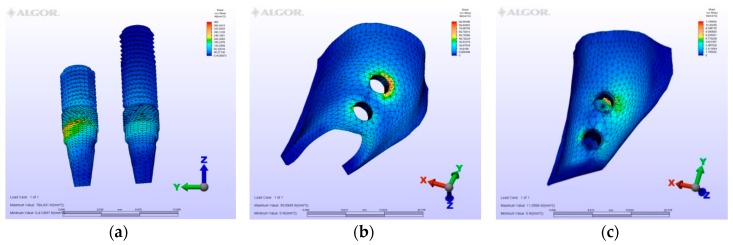
Model M1, oblique loading. (**a**) the maximum von Mises stresses around implants; (**b**) maximum principle stress in cortical bone; (**c**) maximum principle stress in cancellous bone.

**Figure 4:**
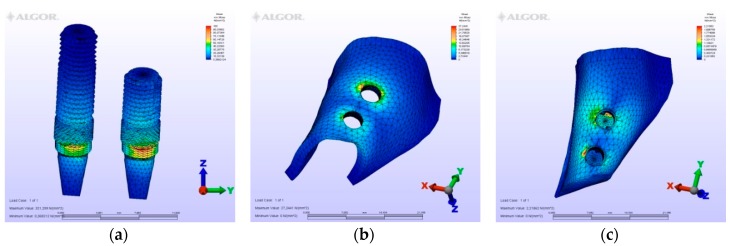
Model M1, horizontal loading. (**a**) the maximum von Mises stresses around implants; (**b**) maximum principle stress in cortical bone; (**c**) maximum principle stress in cancellous bone.

**Figure 5:**
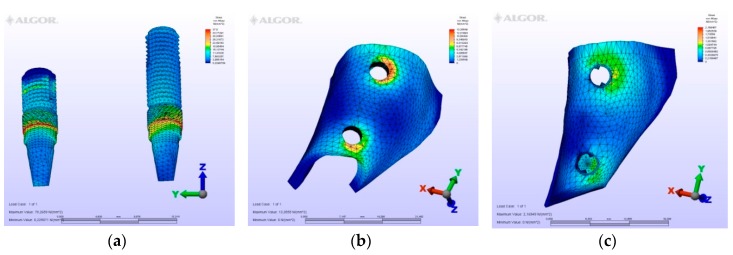
Model M2, axial loading. (**a**) the maximum von Mises stresses around implants; (**b**) maximum principle stress in cortical bone; (**c**) maximum principle stress in cancellous bone.

**Figure 6:**
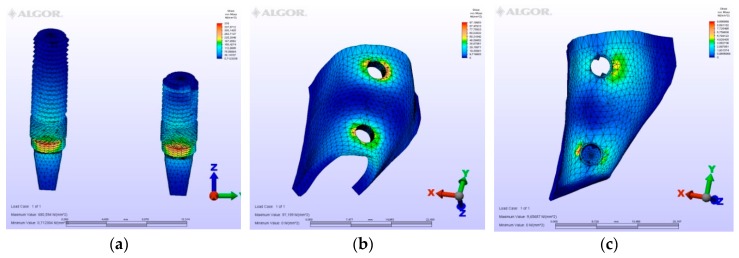
Model M2, oblique loading. (**a**) the maximum von Mises stresses around implants; (**b**) maximum principle stress in cortical bone; (**c**) maximum principle stress in cancellous bone.

**Figure 7:**
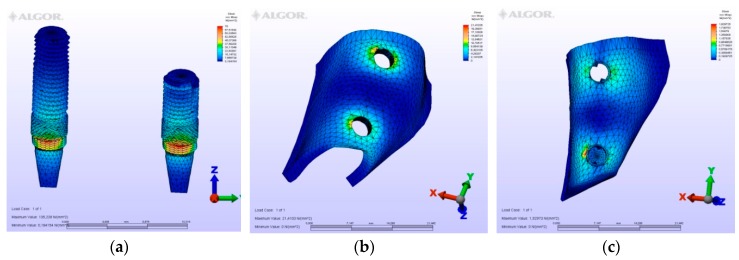
Model M2, horizontal loading. (**a**) the maximum von Mises stresses around implants; (**b**) maximum principle stress in cortical bone; (**c**) maximum principle stress in cancellous bone.

**Figure 8:**
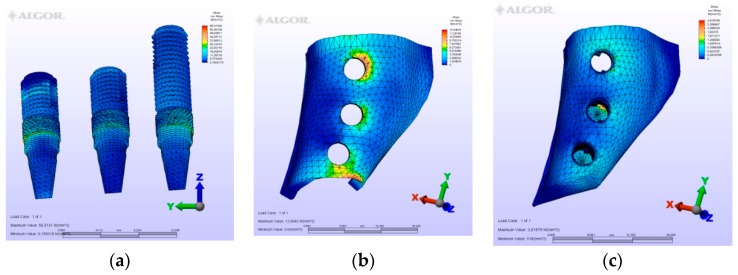
Model M3, axial loading. (**a**) the maximum von Mises stresses around implants; (**b**) maximum principle stress in cortical bone; (**c**) maximum principle stress in cancellous bone.

**Figure 9:**
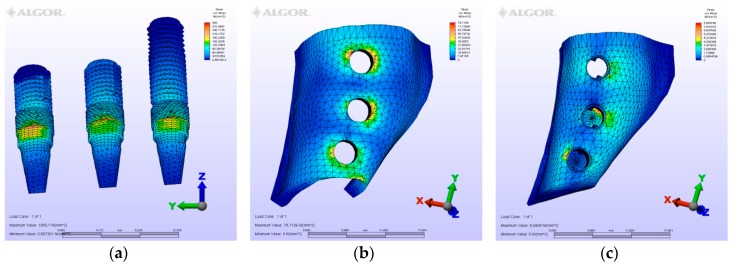
Model M3, oblique loading. (**a**) the maximum von Mises stresses around implants; (**b**) maximum principle stress in cortical bone; (**c**) maximum principle stress in cancellous bone.

**Figure 10:**
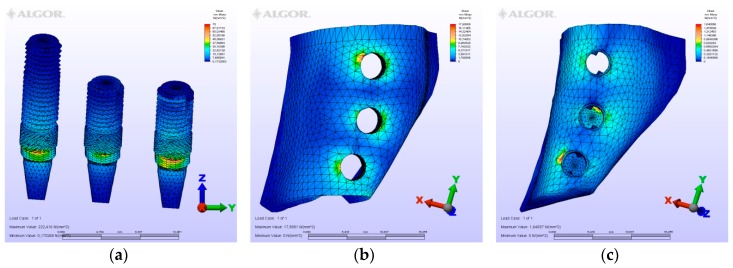
Model M3, horizontal loading. (**a**) the maximum von Mises stresses around implants; (**b**) maximum principle stress in cortical bone; (**c**) maximum principle stress in cancellous bone.

**Figure 11:**
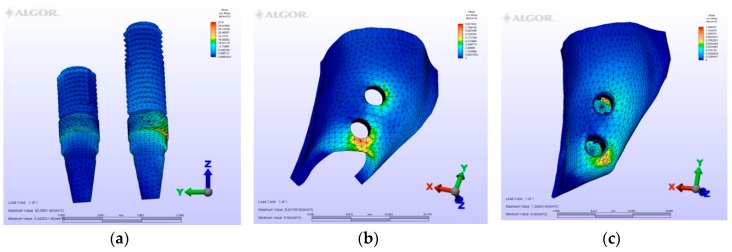
M4, axial loading. (**a**) the maximum von Mises stresses around implants; (**b**) maximum principle stress in cortical bone; (**c**) maximum principle stress in cancellous bone.

**Figure 12:**
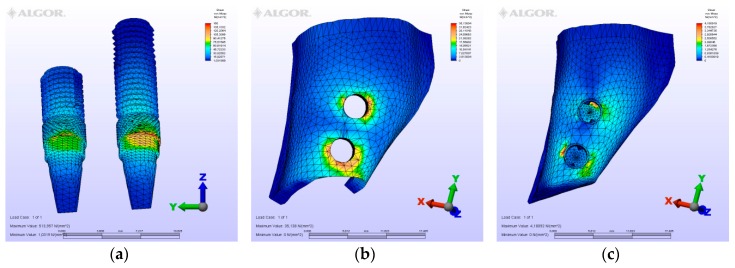
M4, oblique loading. (**a**) the maximum von Mises stresses around implants; (**b**) maximum principle stress in cortical bone; (**c**) maximum principle stress in cancellous bone.

**Figure 13:**
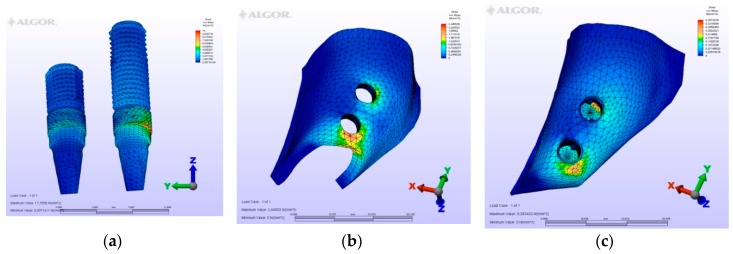
M4, horizontal loading, (**a**) the maximum von Mises stresses around implants; (**b**) maximum principle stress in cortical bone; (**c**) maximum principle stress in cancellous bone.

**Table 1:** Mechanical properties of materials (21).

Material	Young’s Modulus (Gpa)	Poisson’s Ratio
Titanium	110	0.35
Cortical Bone	13.7	0.3
Trabecular Bone (D3)	1.37	0.3
Cr-Co Alloy	218	0.33
Feldspatic Porcelain	82.8	0.35

**Table 2:** Maximum von Mises Stress in Implants (MPa).

Model no	Vertical Force	Horizontal Force	Oblique Force
Model 1	127.32	97.76	360.91
Model 2	79.28	58.34	242.23
Model 3	59.28	44.56	185.68
Model 4	33.88	20.72	105.45

**Table 3:** Minimum–Maximum Principle Stress in Cortical Bone (MPa): Max: maximum principle stress; Min: Minimum principle stress.

Loading type	Vertical Force		Horizontal Force		Oblique Force	
Model no	Minimum Principle Stress	Maximum Principle Stress	Minimum Principle Stress	Maximum Principle Stress	Minimum Principle Stress	Maximum Principle Stress
Model 1	28.37	23.67	23.18	19.99	79.16	67.48
Model 2	16.59	16.48	13.03	13.17	50.77	56.82
Model 3	12.20	12.49	9.34	8.55	39.79	45.57
Model 4	6.05	5.51	4.59	4.93	19.88	23.52

**Table 4:** Minimum–Maximum Principle Stress in Cancellous Bone (MPa): Max: maximum principle stress; Min: Minimum principle stress.

Loading type	Vertical Force		Horizontal Force		Oblique Force	
Model no	Minimum Principle Stress	Maximum Principle Stress	Minimum Principle Stress	Maximum Principle Stress	Minimum Principle Stress	Maximum Principle Stress
Model 1	15.88	3.99	9.79	2.95	25.34	9.77
Model 2	10.19	2.35	5.44	1.87	14.98	5.88
Model 3	6.77	1.89	3.73	1.25	10.37	3.55
Model 4	2.49	1.18	1.23	0.69	4.79	2.35

**Table 5:** Maximum Displacement (mm).

Model No	Vertical Force	Horizontal Force	Oblique Force
Model 1	0.00724	0.00295	0.01998
Model 2	0.00374	0.00183	0.01542
Model 3	0.00302	0.00148	0.01205
Model 4	0.00182	0.00111	0.00595
